# Large-Area Nanogap Platforms for Surface-Enhanced Raman Spectroscopy Toward Sensing Applications: Comparison Between Ag and Au

**DOI:** 10.3390/bios15060369

**Published:** 2025-06-09

**Authors:** Arunkumar Alagurasu, Satyabrat Behera, Joon-Mo Yang, Dai-Sik Kim, Seon Namgung

**Affiliations:** 1 Department of Physics, Ulsan National Institute of Science and Technology, Ulsan 44919, Republic of Korea; arun@unist.ac.kr (A.A.); satyabrat2020@unist.ac.kr (S.B.); daisikkim@unist.ac.kr (D.-S.K.); 2 Department of Biomedical Engineering, Ulsan National Institute of Science and Technology, Ulsan 44919, Republic of Korea; jmyang@unist.ac.kr

**Keywords:** nanogap, Surface Enhanced Raman Spectroscopy (SERS), surface plasmon hotspot, molecule detection, biomedical sensor

## Abstract

Sub-wavelength metallic nanostructures allow the squeezing of light within nanoscale regions, called plasmonic hotspots. Squeezed near-field light has been demonstrated to detect, modulate, and generate light in more effective ways. The enhanced electric field in the plasmonic hotspots are also utilized for identifying molecular fingerprints in a more sensitive manner, i.e., surface-enhanced Raman spectroscopy (SERS). SERS is a versatile tool used to characterize chemicals and biomolecules with the advantages of label-free detection, specificity, and high sensitivity compared to fluorescence and colorimetric sensing methods. With its practical and diverse applications such as biomedical sensing, the evaluation of SERS on diverse nano-structure platforms and materials is highly in demand. Nanogap structures are promising SERS platforms which can be fabricated over a large area with uniform nanoscale gap size. Here, we demonstrate the fabrication of large-area metal–insulator–metal nanogap structures with different metals (i.e., Au and Ag) and analyze material dependence on SERS. While both nanometer-sized gap structures exhibit a large enhancement factor for Raman spectroscopy, Ag-based structures exhibit 58- and 15-times-larger enhancement factors for bottom and top plasmonic hotspots, respectively. The enhanced detection on a silver nanogap platform is attributed to enhanced electric field in the gap, as confirmed by simulation. Our findings provide not only a way to better understand SERS in different metallic nano platforms but also insights for designing highly sensitive nanoscale chemical and biomedical sensors.

## 1. Introduction

Metallic nanostructures excite surface plasmons, which are the collective oscillation of free electrons, and thereby strongly interact with incident light, eventually increasing absorption or scattering the cross-section of light [1]. Plasmonic nanostructures have been extensively utilized for development in diverse research and industrial fields, such as modern telecommunications, quantum photonics, and highly sensitive sensors, in revolutionary ways [2,3,4,5]. These subwavelength nanostructures are integrated with nanodevices to improve imaging, enhance signal integration, and extend the sensing detection limit [6,7,8,9]. Noble metals including gold (Au) and silver (Ag) are widely used for plasmonic nanostructures due to their dielectric functions which support plasmons in the visible to near-infrared range with low optical loss and chemical stability [10,11,12]. The plasmonic oscillations can be confined within subwavelength space with a highly enhanced electric field, called plasmonic ‘hotspot’ [13]. Plasmonic hotspots have been also employed for Raman measurement. Ever since the discovery of the Raman scattering phenomenon, this technique has been effectively used to study molecular interactions and identify molecular fingerprints of probe molecules through their interaction with incident photons. These photons undergo both elastic and inelastic scattering; however, the intensity of the inelastic scattering is significantly low (about 10^−7^), which limits the practical application. To address this limitation, several advanced techniques have been developed to increase the number of photons from inelastic scattering. One of the most effective approaches involves the utilization of metallic nanostructures to amplify Raman signals using plasmonic hotspots, i.e., surface-enhanced Raman spectroscopy (SERS), which enables highly sensitive molecular detection. Researchers are now focusing on developing ultrasensitive SERS platforms for commercial and real-world applications. Among recent innovations, the use of metasurfaces has shown great potential by providing high reproducibility and spatially uniform Raman signal enhancement, improving overall sensing performance [14,15]. 

Metal–insulator–metal (MIM) nanostructures, such as nanoparticle-on-mirror (NPoM), aggregations of nanoparticles, nanopillars, and resonant metasurfaces [16,17,18], have attracted lot of attention due to strongly confined plasmonic hotspots. The hotspots in the nanostructures are employed to increase the Raman intensity, since the enhanced electric field of the hotspots increases the Raman scattering rate and chemical reactions occurring therein [13]. Thus, MIM nanostructures are a promising platform to detect molecular fingerprints with high sensitivity for practical applications such as biomedical sensors. MIM plasmonic platforms can be fabricated by modern and conventional semiconductor fabrication processes such as photolithography, electron beam lithography (EBL), and focused ion beam (FIB) lithography [19,20,21,22,23]. However, most of these structures suffer from non-uniformity of structure and inability to be massively fabricated. To overcome these limitation, large-area nanogap platforms have been developed, in which the gap size is precisely defined by the atomic layer deposition method [24]. Large-area nanogap platforms have been utilized for various applications such as solar energy conversion, terahertz-based biosensor, adhesion lithography-based memristive devices, non-contact moisture sensing, and strain sensors [25,26,27,28,29]. Recently, a large-area nanogap platform has been also utilized for SERS, demonstrating an active flexible nanogap used to modulate the molecular vibrational mode, temperature-tuned gap width for advanced molecule detection, dielectrophoretic trapping of biological molecules, and electrotunable plasmonic hotspots for effective sensing [1,25,26,27,28,29,30,31,32].

Since this nanogap platform can be fabricated on a wafer scale with a uniform gap size, it offers a significant advantage for manufacturing reliable sensing devices with consistent performance over a large area. This consistency is essential for practical applications in chemical and biological sensing, where sensitivity, reliability, and scalability are critical requirements. Thus, for broader sensing applications, SERS studies using large-area nanogap platforms are strongly in demand. In this paper, we demonstrate the fabrication of nanogap platforms with two representative noble metals, i.e., Au and Ag, and systematically study and compare the SERS performance of both structures in detecting the molecular fingerprints of chemicals and biomolecules. With the plasmonic hotpots, the enhanced Raman signals of diverse molecules are detected in the nanogap platforms. We reveal the different locations of plasmonic hotspots and their electric field enhancement in both structures with different metals using simulation based on the finite element method. Of note, our results clearly show different electric field enhancement at the hotspots in the nanogap geometry depending on the metals. Our results provide important insights for designing nanoscale plasmonic structures as highly sensitive SERS platforms for promising chemical and biomedical sensors.

## 2. Materials and Methods

### 2.1. Fabrication of Nanogap

We cleaned 300 nm SiO_2_/Si substrates in acetone and isopropyl alcohol in an ultrasonication bath. For the fabrication of Au nanogap structures, we then deposited a 100 nm thick gold film with 3 nm Ti as an adhesion layer using an electron beam evaporator (KVE-E2000, Korea Vacuum Tech, Gimpo, Republic of Korea) on the cleaned substrate. Further, we introduced the sacrificial layer on the Au film through the conventional photolithography process, which acts as a hard mask. Then, we performed the Ar^+^ gas ion milling process to etch out the exposed gold film. Following that, we used the ALD system (Atomic basic, CN1 Co., Ltd., Hwaseong, Republic of Korea) to deposit Al_2_O_3_ uniformly over a large area. The second layer of gold thin film was deposited on the sample to form the MIM geometry. Then, again, ion milling processes were carried out at an oblique angle to etch the excess thick gold film on top of the sacrificial layer. Following this, chemical etching processes were undertaken in a KOH environment at room temperature to remove the insulator layer (Al_2_O_3_), which defines the metal–air gap–metal geometry. Finally, the sacrificial layer was removed through a chemical etching process. The same process was used to fabricate the Ag nanogap nanostructures with Ag deposition.

### 2.2. Preparation of Molecules

All the chemicals and materials were purchased and used as received without further purification. The probe molecules of Rhodamine 6G (R6G) (CAS No. 989-38-8, Tokyo Chemical Industry, Tokyo, Japan) and Rhodamine B (RhB) (CAS No. 81-88-9, Alfa Aesar, Haverhill, MA, USA) powder were dissolved in ethanol with 1 mM concentration. Nile Blue A (NBA) (CAS No. 3625-57-8, Sigma-Aldrich, St. Louis, MI, USA) and glucose (CAS No. 50-99-7, Sigma-Aldrich, St. Louis, MI, USA) were dissolved in deionized water. The probe molecules were drop-cast on the nanogap region.

### 2.3. SERS Measurements

SERS measurements were performed in a confocal Raman spectrometer (Alpha300R, WITec, Ulm, Germany) with 532 nm laser as a source of excitation. We tightly focused the laser on the nanogap using the 50× objective lens (NA = 0.5), with a defined laser spot size of ~1 μm. The power of the excitation laser source was kept at 5 mW throughout the experiment, with a 1 s exposure time. To ensure the stability of the Raman signal, we recorded the Raman spectrum with 10 accumulations. All the reported Raman peaks were normalized in OriginPro 9.8 software (OriginLab Corporation, Northampton, MA, USA).

### 2.4. Numerical Simulation

COMSOL Multiphysics 6.2 (COMSOL Inc., Burlington, MA, USA) was used with wave optics module for electromagnetic simulation. The structure length was set to 400 nm and the nanogap was evaluated at the middle of the structure. The nanogap width and height were fixed at 20 nm and 100 nm, respectively. A plane wave source with electric field polarized along the *x*-axis and propagating along the *y*-axis was introduced. Scattering boundary conditions were implemented to minimize reflections at the boundaries. Refractive index values for Ag, Au and SiO_2_ were obtained from reference [33].

## 3. Results and Discussion

### 3.1. Mass Fabrication of Nanogap Platform

**Figure 1:**
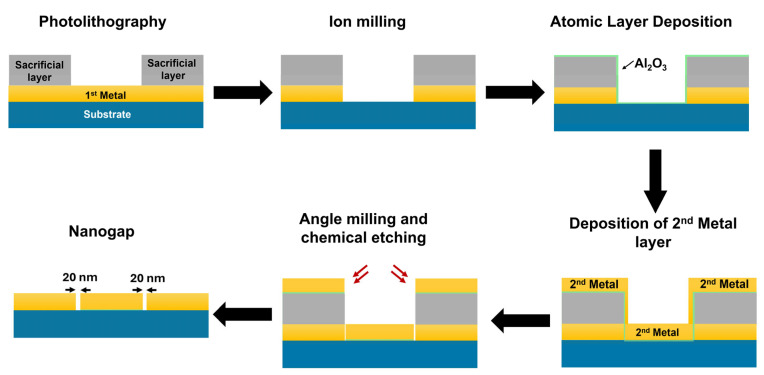
Schematics of the mass fabrication of a nanogap platform on a wafer scale (not to scale).

Figure 1 shows the fabrication process of the nanogap platform for SERS analysis. We used a 4-inch SiO_2_/Si wafer for the nanogap fabrication. For the fabrication of Au nanogap platforms, the process began with the deposition of the first layer of Au thin film (100 nm) under high vacuum (2 × 10^−6^ Torr) with the rate of deposition (0.5 Å/s) and used Ti as an adhesion layer to prevent peel-off during the lift-off process [34]. We used the conventional photolithography method to assign the required geometry to the nanogap structure. Here, we used wafer-scale slit patterns for the nanogap geometry and then deposited a sacrificial layer of vanadium on it as a hard mask. After lift-off, we used an Ar^+^ gas ion miller to etch out the exposed part of the Au film. Following that, a 20 nm insulating layer (Al_2_O_3_) was deposited uniformly on the wafer. The thickness of the deposited Al_2_O_3_ layer defines the gap size of our nanogap structures. The second layer of Au was deposited on top of the entire wafer, and the ion milling process was performed again at a 80-degree tilt-angle to remove the excess Au. Further, the wafer was immersed in KOH solution to remove the insulating layer to open the air gap. The fabrication process was completed with the etching of the sacrificial layer using chromium etchant. For the Ag nanogap platforms, we used the same process with Ag deposition. Notably, here, due to the chemical incompatibility of silver, the photoresist was implemented as a hard mask instead of vanadium metal [19]. Through this process, one can fabricate nanostructures on a full wafer scale.

**Figure 2:**
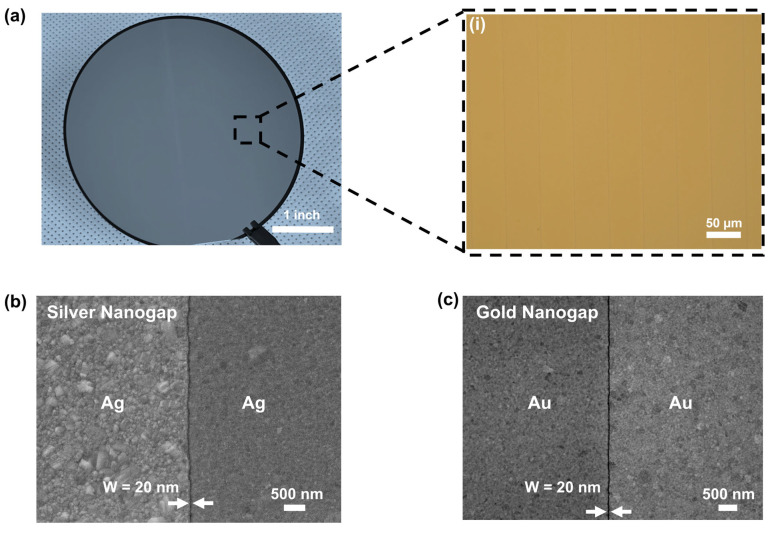
Characterization of nanogap platforms. (**a**) Photograph image of Ag nanogap structures fabricated on a 4-inch wafer. (i) Optical microscopy image of the enlarged view of our nanogap platform. Top-view FESEM images of (**b**) Ag and (**c**) Au nanogap with uniform 20 nm width.

Figure 2a shows a digital photograph image of nanogap structures fabricated with Ag on a SiO_2_/Si wafer. An enlarged image (i) obtained using optical microscopy represents the wafer-scale long nanogap structures with slit patterns. The wafer-scale fabrication of uniform nanogap structures is beneficial for mass production of SERS platforms for practical applications. Figure 2b,c show the field effect scanning electron microscopy (FESEM) images of nanogap structures fabricated with silver and gold, respectively. The uniform 20 nm-wide nanogap between the first and second deposited metal layers are clearly observed. This uniformity of nanostructures over the wafer scale should allow reliable measurement of SERS on nanogap devices from different locations on a wafer.

### 3.2. SERS Measurement

**Figure 3:**
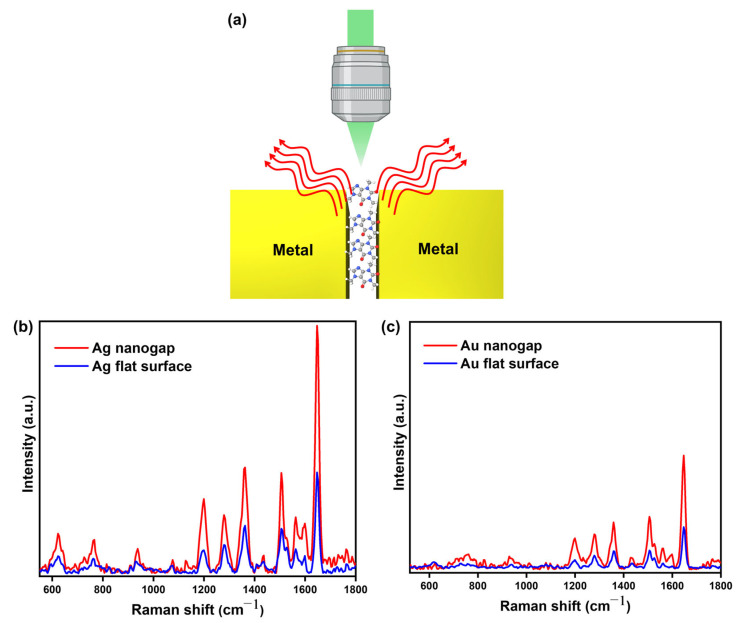
SERS analysis of the fabricated nanogap platform. (**a**) Schematic representation of SERS measurement. (**b**) Raman peaks of RhB molecules from the Ag flat surface and nanogap region. (**c**) Raman peaks of RhB molecules from the Au flat surface and nanogap region.

Figure 3a illustrates the schematic of the SERS measurement setup using the nanogap platform. Raman signals were collected from both nanogap structures and flat metal surfaces, which were coated with RhB molecules. A simple and effective drop-casting method was used to deposit the probe molecules onto the nanogap samples, followed by drying at ambient temperature for 30 min to ensure proper adhesion. A 532 nm laser was then focused onto the nanogap structures, which were fabricated using either silver (Ag) or gold (Au). The resulting Raman signals from the RhB molecules were measured to evaluate the SERS performance of each nanogap configuration. This setup allowed direct comparison of the enhancement effects between nanogap and flat surface structures for both metals. Figure 3b,c show the Raman peaks for RhB measured on the flat surface and the nanogap regions of Ag and Au platforms, respectively. The enhanced Raman signals were observed in nanogap structures compared the ones in the bare flat region for both of Ag and Au cases, which is attributed to the formation of plasmonic hotspots in the nanogap structures and increased Raman scattering therein, i.e., SERS. We measured five times and calculated the ratio between the Raman signals of the RhB molecules (Supplementary Information Figure S1). For instance, for the aromatic stretching vibrational peak of RhB located at 1648 cm^−1^, the ratio of the average signal of the Ag nanogap region over the Ag flat region and that of the Au nanogap region over the Au flat region is 1.824 and 2.355, respectively. The ratio of the signal of the Ag nanogap over the Au nanogap is 5.211. We also measured and calculated the average signal enhancement ratio of the nanogap region over the flat region for Ag and Au cases for different modes at 1195 cm^−1^ (C-H in-plane bending mode) and 1280 cm^−1^ (H-C-H wagging vibration mode) (Supplementary Information Figure S1). The average enhancement ratio of the Ag nanogap region over the Ag flat region for 1195 cm^−1^ and for 1280 cm^−1^ is measured as 2.016 and 1.978, respectively, which is similar to the enhancement ratio for 1648 cm^−1^. For the Au cases, the average enhancement ratio of the Au nanogap region over the Au flat region for 1195 cm^−1^ and for 1280 cm^−1^ is measured as 2.763 and 2.849, respectively, which is also similar to the enhancement ratio for 1648 cm^−1^_._ Thus, there is little mode-specific Raman enhancement of the molecules in our nanogap platform. The observed stronger Raman signal from the Ag nanogap structures compared to the signals from Au ones is attributed to the higher electric field concentrated within the Ag nanogap structures [35,36].

### 3.3. Numerical Simulation

**Figure 4:**
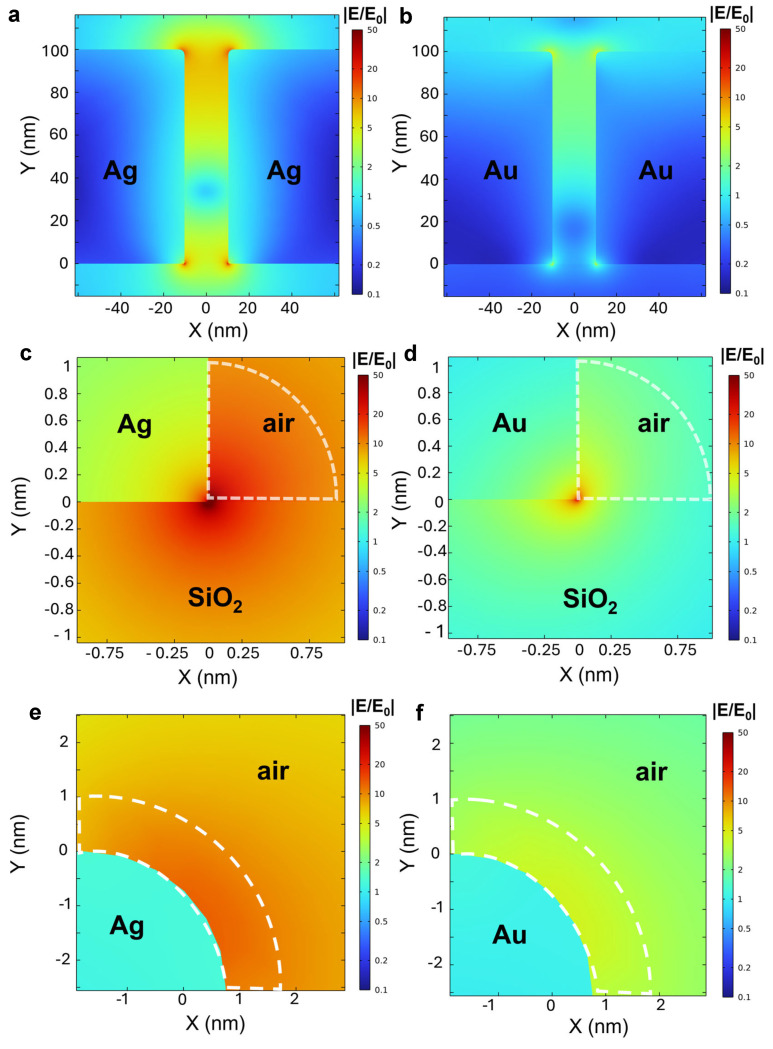
Finite element analysis of electric field distribution in (**a**) Ag and (**b**) Au nanogap structures, respectively. Zoomed-in images of plasmonic hotspots from the bottom (**c**,**d**) and top (**e**,**f**) parts of (**a**,**b**). The ratio of E-field in the dashed line area is averaged to obtain the average enhancement in E-field in the main text.

To further study the SERS on our nanogap structures, finite element method simulation is performed as shown in Figure 4 (see Section 2.4 for simulation details). We study the hotspot formation within the gap region and their electric field distribution across the free space. The cross-sectional electric field distribution for Ag and Au nanogaps is shown in Figure 4a and Figure 4b, respectively. Of note, the color scales of the images are based on the logarithmic scale. Plasmonic hotspots with the concentrated electric field are formed at the edge of the metal/air interface at the *top* part of the structure and at the metal/SiO_2_/air interface at the *bottom* part of the structure for both cases. The electric field of the plasmonic hotspots in Ag nanogap platform is stronger than that in the Au nanogap platform. Of interest, the electric field of the plasmonic hotspot at the *top* part is stronger for the Au case, while at the *bottom* part, it is stronger for the Ag case. The clear differences in the intensity of the electric field at the plasmonic hotspots of both structures are shown in the zoomed-in images (Figure 4c–f). The average value of the electric field enhancement in the area (enclosed in dashed line) within 1 nm from the *bottom* hotspot is 7.557 and 2.737 for Ag and Au nanogaps, respectively. On the other hand, the average value of the electric field enhancement in the area (enclosed in dashed line) within 1 nm from the *top* hotspot is 6.098 and 3.113 for Ag and Au nanogaps, respectively. For the *top* hotspot case, the larger electric field is formed in the Au nanogap, which is contrary to the *bottom* hotspot case. Further, we calculate the SERS enhancement factor (EF) of our platforms, which effectively elucidates the fundamental mechanism of SERS [37]. The calculated SERS EF is given by the following equation [18].

$$EF={\left|E_{hotspot}/E_{0}\right|}^{4}\times \frac{V_{total}}{V_{hotspot}}$$

In this formula, *E_hotspot_*/*E_0_*, *V_total_*, and *V_hotspot_* represent the ratio of the enhanced electric field in the nanogap hotspot region, the total volume of the nanogaps in the focused laser beam spot, and the volume of the hotspot, respectively. The calculated EF is 8.305 × 10^6^ and 1.428 × 10^5^ for the *bottom* hotspots in the Ag and Au nanogap structures, respectively, exhibiting a 58-times-larger EF for the Ag nanogap as compared to the Au nanogap. The calculated EF is 1.174 × 10^6^ and 7.966 × 10^4^ for the *top* hotspots in the Ag and Au nanogap structures, respectively, exhibiting a 15-times-larger EF for the Ag nanogap as compared to the Au nanogap. Altogether, the top and bottom parts of the Ag nanogap create a strong and well-defined electromagnetic environment which improves the sensitivity of the sensing medium and makes it more effective for detecting molecules. The larger SERS signal on the Ag platform is attributed to the stronger electric field formed at the MIM nanogap structure for the Ag case [38]. For optimized design for SERS using a large-area nanogap platform, we also performed numerical simulations on 50 nm thick Ag and Au nanogap structures (Supplementary Information Figure S2). The average value of electric field enhancement from the *bottom* hotspot is 5.9611 and 3.9476 for the Ag and Au nanogaps, respectively. On the other hand, the average value of the electric field enhancement in the *top* hotspot is 0.7449 and 1.8971 for Ag and Au nanogaps, respectively. These values are smaller than the values of 100 nm thick nanogaps, except for the bottom hotspot of the Au nanogap. Thus, 100 nm thick Ag nanogap samples should exhibit larger Raman signals for molecule sensing. Of interest, the averaged electric field on the *top* hotspot in the Ag nanogap is 8 times larger for the 100 nm thick Ag nanogap, corresponding to a 4500-times-larger EF, allowing more sensitive molecule detection with easy access to the *top* hotspot region. Thus, the thickness of the nanogap should be also considered as a crucial factor in designing SERS-based molecular sensors with our nanogap platform. We also performed numerical simulations with different excitation laser wavelengths of 633 nm and 785 nm (Supplementary Information Figure S3). With an excitation wavelength of 633 nm, the average value of electric field enhancement from the *bottom* hotspot is 10.799 and 14.797 for the Ag and Au nanogaps, respectively. On the other hand, the average value of the electric field enhancement in the *top* hotspot is 6.1228 and 9.6569 for the Ag and Au nanogaps, respectively. Those values are larger than those with 532 nm excitation. Additionally, with an excitation wavelength of 785 nm, the average electric field from the *bottom* of the nanogap was calculated to be 4.4935 for Ag and 5.3326 for Au, while the corresponding values from the *top* part were 1.0513 for Ag and 1.5394 for Au, respectively. Thus, with excitation wavelengths of 633 nm and 785 nm, the Au nanogap should exhibit a higher SERS signal than the Ag one. This is presumably attributed to the stronger absorption at 532 nm for Au. Therefore, the optimal selection of excitation wavelength and material for the nanogap should be considered in designing high-performance molecular sensors based on SERS.

### 3.4. Versatile SERS Application of Nanogap Structures

**Figure 5:**
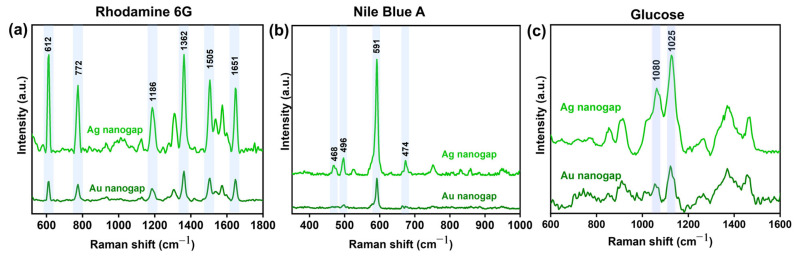
Versatile application of our nanogap platform to measure SERS. SERS peaks from (**a**) Rhodamine 6G, (**b**) Nile Blue A, and (**c**) glucose biomolecules measured on Ag and Au nanogap platforms. Ag nanogap platforms exhibit larger SERS signals than Au ones.

To further confirm the higher sensitivity of the Ag nanogap platform shown in Figure 3 and demonstrate the application of our SERS platform on different molecules, we conducted SERS measurements using various chemicals and biomolecules. Figure 5 shows the Raman peaks of R6G, NBA, and glucose molecules from both Ag and Au nanogap structures. We observed clear molecular fingerprints of the probe molecules on both Ag and Au nanogap structures. In all cases, the Ag nanogap structures produced significantly stronger SERS signals compared to their Au counterparts. This enhanced performance demonstrates that Ag-based nanogap platforms offer higher sensitivity, making them a more suitable choice for developing advanced chemical and biological sensors based on SERS technology for reliable and efficient molecular detection. Distinct Raman peaks at 612 cm^−1^, 1362 cm^−1^, and 1651 cm^−1^ with the R6G molecule were detected using our nanogap platform, especially with the Ag nanogap platforms. Additionally, the NBA was also detected with a higher Raman signal at the distinct peak at 591 cm^−1^ on our Ag nanogap platform. Of particular interest, we identified and traced the characteristic Raman peaks at 1060 cm^−1^ and 1125 cm^−1^ for glucose, which are crucial for monitoring glucose levels in the human body (Figure 5c) [39]. The strong and consistent SERS signals of glucose suggest that our nanogap platform holds significant promise for biomedical sensing applications, especially for non-invasive glucose detection and health-related diagnostic tools.

## 4. Conclusions

In summary, to design a highly sensitive molecular detection platform based on large-scale nanogap structures, we studied and compared the performance of SERS with the nanogap structures fabricated with two representative noble metals (i.e., Ag and Au). We demonstrate the fabrication of wafer-scale nanogap platforms based on the thin insulating layer with two representative noble metals of Ag and Au. Stronger Raman signals were observed on both nanogap platforms compared to the flat surface of the same materials, indicating that our nanogap structures are promising as molecular sensing platforms based on SERS. In addition, the Ag nanogap platform exhibits stronger Raman signals than Au one with the excitation wavelength of 532 nm. For in-depth study of the stronger SERS signal on the Ag nanogap platform, we performed finite element method simulation on both nanogap platforms. The relative electric field enhancement of the plasmonic hotspots depends on the materials used to fabricate the nanogap structures, and the Ag nanogap platform exhibits stronger EF of SERS than the Au nanogap platform with the given experimental conditions. With additional simulation with different thickness of the nanogap structure and excitation wavelength, we found different field enhancement for the top and bottom hotspots. This result provides crucial insights for optimized selection of materials, geometric parameters, and excitation wavelength to design-high performance molecular sensing devices based on SERS using our large-area nanogap platform. Finally, we demonstrate that our SERS platform is applicable for diverse chemicals and biomolecules detection and confirm the consistently higher sensitivity of the Ag nanogap platform for the given experimental conditions. Our study provides a deeper understanding of SERS in nanogap structures from the material point of view and a crucial strategy to develop more-sensitive SERS devices for chemical and biomedical sensing applications.

## Data Availability

Inquiry can be direct to the corresponding author.

## Supplementary Materials

The following supporting information can be downloaded at: https://www.mdpi.com/article/10.3390/bios15060369/s1, Figure S1: Raman signal at different modes; Figure S2: Electric field simulations for 50 nm thickness; Figure S3: Electric field simulations with different wavelengths.
