# Supplementary material for: Large-Area Nanogap Platforms for Surface-Enhanced Raman Spectroscopy Toward Sensing Applications: Comparison Between Ag and Au
Source: Biosensors (Basel). 2025 Jun 9;15(6):369. doi: 10.3390/bios15060369 (PMC12191043; doi:10.3390/bios15060369)
Supplement: Supplementary file 1 [file biosensors-15-00369-s001.zip › biosensors-3647973-supplementary.pdf]

Supplementary Information

Large-Area Nanogap Platforms for Surface-Enhanced Raman Spectroscopy Toward Sensing Applications: Comparison Between Ag and Au

Arunkumar Alagurasu [1], Satyabrat Behera [1], Joon-Mo Yang [2], Dai-Sik Kim [1] and Seon Namgung [1]*

1 Department of Physics, Ulsan National Institute of Science and Technology, Ulsan 44919, Republic of Korea; arun@unist.ac.kr (A.A.); satyabrat2020@unist.ac.kr (S.B.); daisikkim@unist.ac.kr (D.-S.K.)
2 Department of Biomedical Engineering, Ulsan National Institute of Science and Technology, Ulsan 44919, Republic of Korea; jmyang@unist.ac.kr
* Correspondence: seon@unist.ac.kr

1. Raman signals at different modes

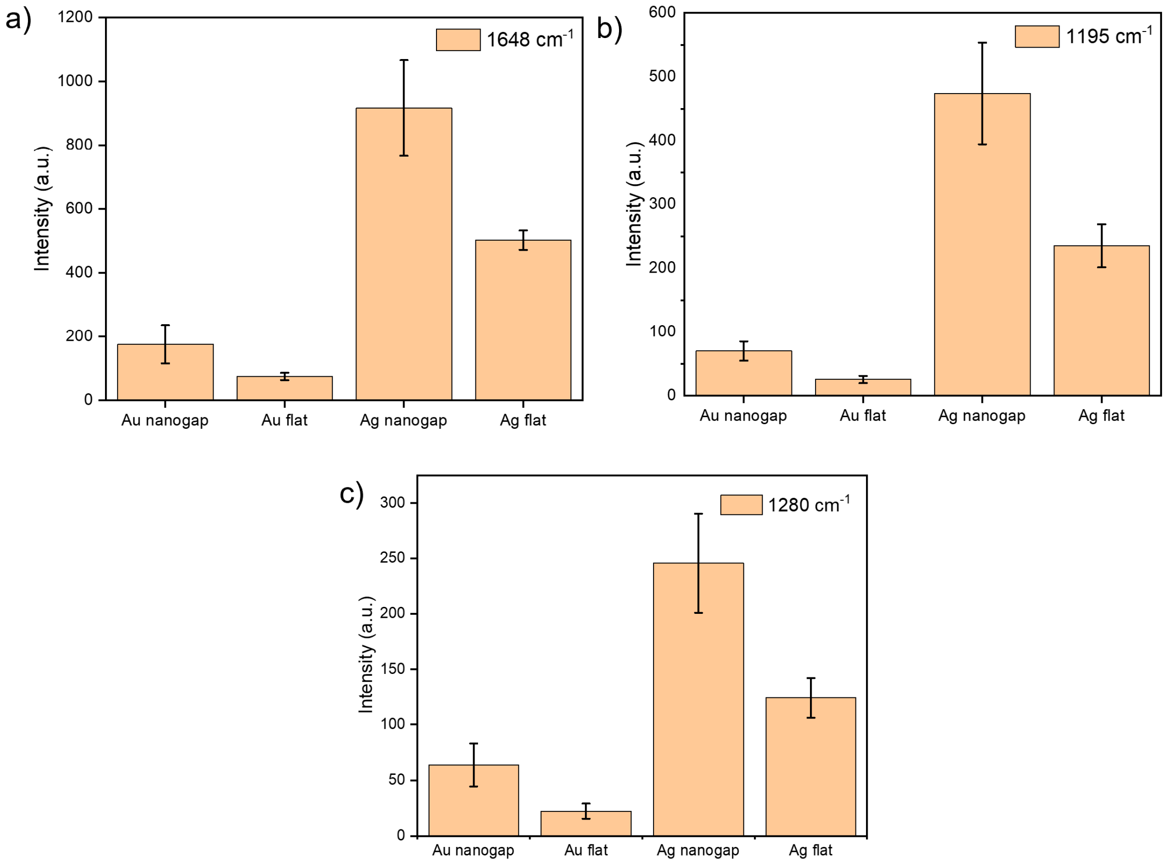

Figure S1. Raman signals for a) 1648 cm^{-1} mode, b) 1195 cm^{-1} mode c) 1280 cm^{-1} mode with error bar.

2. Electric field simulations for 50 nm thickness

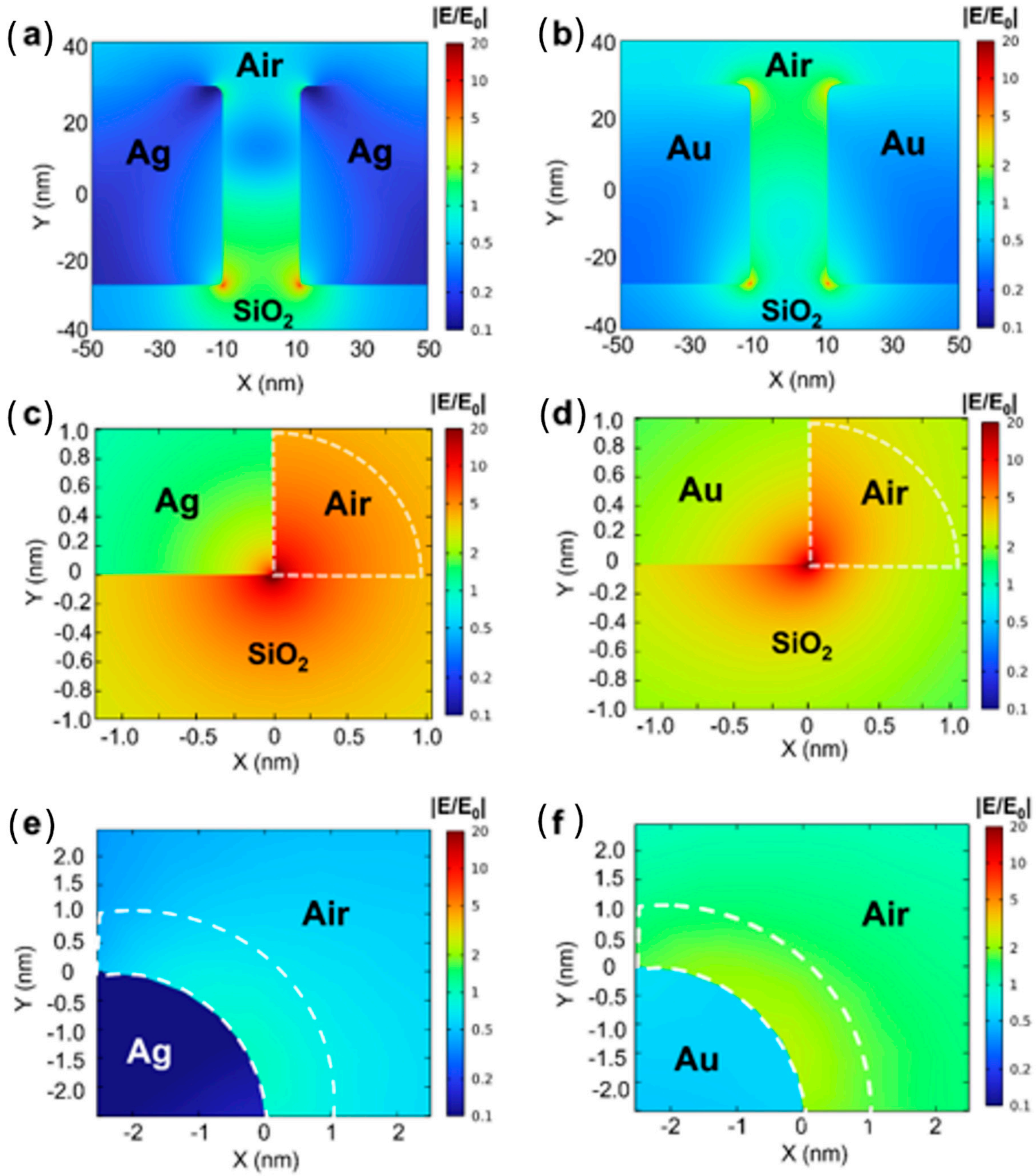

Figure S2. Finite element analysis of electric field distribution in a) Ag and b) Au nanogap structures, respectively. Zoom-in images of plasmonic hotspots from bottom (c, d) and top (e.f) parts of Figure a and b.

3. Electric field simulations with different wavelengths

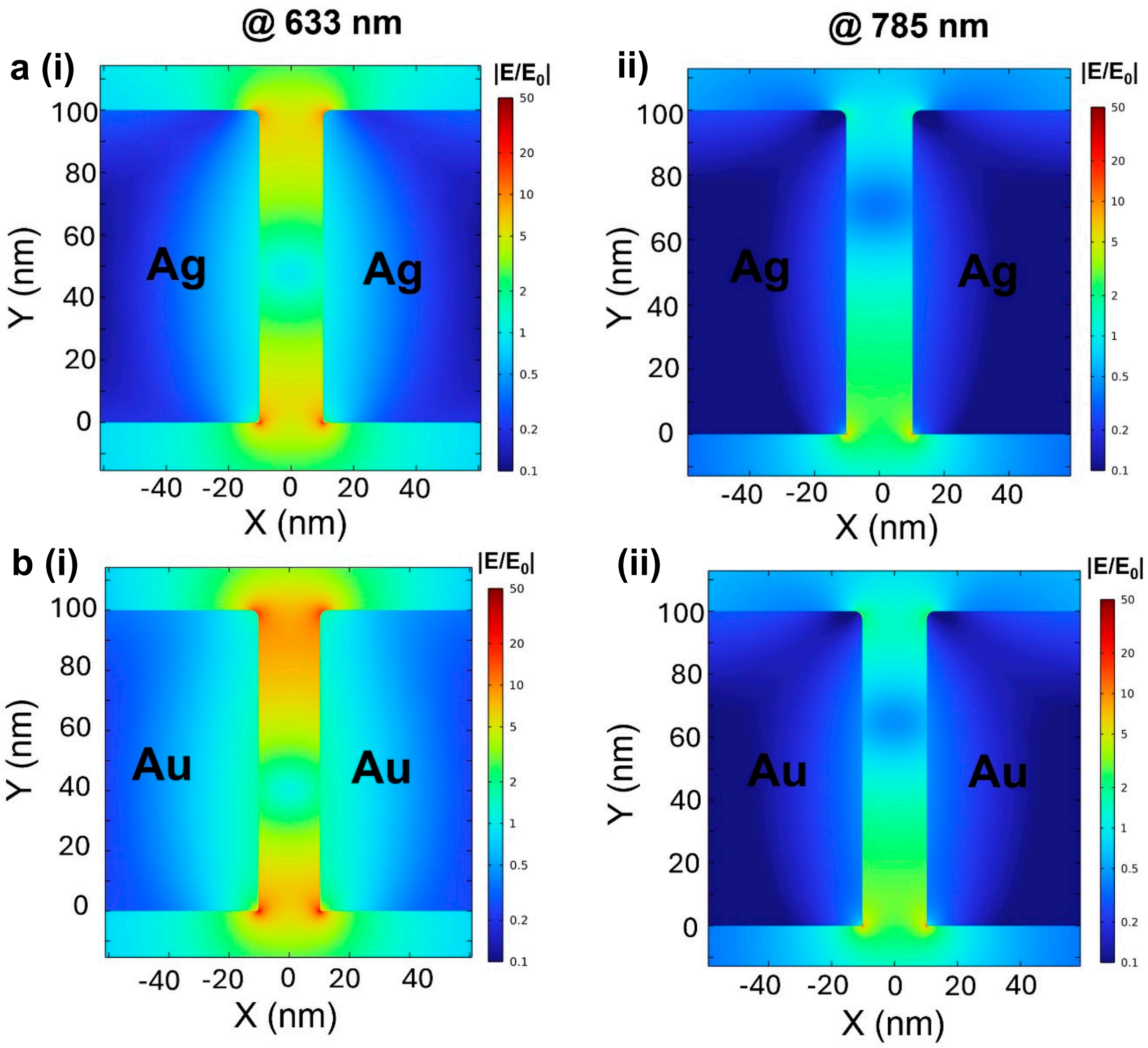

Figure S3. Finite element analysis of both Ag and Au nanogaps at various wavelengths. Silver nanogap structure; a (i) at 633 nm, (ii) 785 nm. Gold nanogap; b (i) 633 nm, (ii) 785 nm
